# It’s X-Related: Biological Bases of Increased COVID-19 Morbidity and Mortality in Men

**DOI:** 10.1210/jendso/bvaa133

**Published:** 2020-09-09

**Authors:** Philip K Angelides, Ishita Jindal, Lefkothea Karaviti, Mitchell E Geffner

**Affiliations:** 1 The Perelman School of Medicine, University of Pennsylvania, Philadelphia, Pennsylvania; 2 Division of Pediatric Endocrinology and Diabetes, Texas Children’s Hospital, Baylor College of Medicine, Houston, Texas; 3 The Saban Research Institute, Children’s Hospital Los Angeles, Keck School of Medicine of the University of Southern California, Los Angeles, California

**Keywords:** androgen receptor, X chromosome, SARS-CoV-2, COVID-19, sex differences

Coronavirus disease 2019 (COVID-19) is caused by severe acute respiratory syndrome coronavirus 2 (SARS-CoV-2). COVID-19 has exhibited impressive, sex-specific differences in disease severity, with males experiencing higher fatality rates than females [1]. Several hypotheses have been proposed to account for these sex differences, including X-linked genes that confer immunoprotection, androgen-receptor activity, and sex- and gender-related social factors that are more common in men than women, including high smoking rates, delays in seeking medical care, and a high prevalence of comorbidities [1].

Biological factors may be responsible, at least in part, for a stronger immune response to COVID-19 infection in females compared to males. The X chromosome contains a number of immune-related genes, including several that are involved in inflammation and are responsible for the innate and adaptive immune responses to infection. Females are functional mosaics for the genes located on the X chromosome, whereas males are hemizygous. Thus, the difference in copy number of X-linked genes involved in the immune response and the presence of genes dedicated to disease susceptibility in females vs males might account for the immune advantage in females [2].

In females, estrogen signaling might also play a protective role in preventing lethal SARS-CoV infections. In a mouse model infected with SARS-CoV, orchiectomy did not affect the disease outcome in male mice, but ovariectomy or estrogen-receptor antagonist treatment led to increased mortality in females [3]. This observation strongly supported the notion that females have stronger innate and adaptive immune responses than males; thus, females are generally more resistant to viral infections than males.

Androgens modulate the immune system by suppressing inflammatory immune cells and the antibody response to infection. This sex-related immunomodulation makes females more prone to autoimmune diseases than males. However, androgen-induced immunosuppression likely contributes to the observation that SARS-CoV-2 infections lead to worse clinical outcomes in males than in females.

Another possible mechanism underlying the male-female discordance in COVID-19 mortality involves the role of the androgen receptor, the gene for which is located on the X chromosome. The transmembrane protease serine 2 co-receptor gene (*TMPRSS2*) encodes a transmembrane serine protease, which is predominantly expressed in the prostate. In lung and prostate cancer cells, *TMPRSS2* expression is androgen dependent [4]. *TMPRSS2* expression is upregulated by androgen-receptor gene polymorphisms and by shortened CAG repeat segments in the androgen-receptor gene [5]. Among the transmembrane proteases that interact with SARS-CoV-2, TMPRSS2 is considered essential for viral pathogenesis. Indeed, before SARS-CoV-2 can enter human cells, its spike protein must be proteolytically cleaved by TMPRSS2. Moreover, the protease might also enhance viral entry by cleaving angiotensin-converting enzyme II (ACE2). ACE2 facilitates the binding of SARS-CoV-2 to the cell, and its gene expression is also upregulated by androgens; thus, *ACE2* expression is higher in males than in females [6]. TMPRSS2 protease inhibitors, such as camostat mesylate, are being investigated as potential therapies for treating COVID-19.

Further evidence in support of an association between the androgen receptor and SARS-CoV-2 pathogenesis is based on the physiology and genetics of hyperandrogenic and hypoandrogenic states. Benign prostatic hyperplasia and prostate cancer are fueled by androgens that also facilitate the expression of *TMPRSS2.* Previous studies have reported an inverse relationship between the number of CAG repeats in the androgen-receptor gene and the level of androgen-receptor transcriptional activity; a shorter CAG repeat length in the first exon of the androgen-receptor gene is associated with androgen hypersensitivity. Shorter CAG repeat lengths also confer vulnerability to developing androgenic alopecia, which has been reported to occur with heightened frequency in men admitted to the hospital with SARS-CoV-2 compared to the general population [7]. Another condition marked by hyperandrogenism is polycystic ovarian syndrome (PCOS), a common endocrine disorder that affects women. PCOS is associated with obesity and metabolic syndrome, which are major risk factors for SARS-CoV-2 lethality [1]. Increased frequency of short CAG repeat length has also been reported in some White, Chinese, and Indian PCOS populations [8]. Unfortunately, no published data are available on the prevalence of PCOS in patients with SARS-CoV-2 infections.

Prepubertal children appear to have a reduced risk of acquiring SARS-CoV-2. Prior to puberty, androgen-receptor expression is low. The Chinese Center for Disease Control and Prevention reviewed 72 314 COVID-19 cases and found that children younger than 10 years comprised less than 1% of the total number of cases, with no reported fatalities. In the United States, the Centers for Disease Control and Prevention reported that, as of April 2020, among the 2572 children with SARS-CoV-2 infections, 15% were younger than 1 year, 26% were age 1 to 9 years, 27% were age 10 to 14 years, and 32% were age 15 to 17 years.

The pathophysiology of morbidity secondary to SARS-CoV-2 may be linked to the heterogeneity of the androgen-receptor gene. Whether or not there are polymorphisms within androgen-receptor genes that differ between men and women, the differences in COVID-19 outcome by sex are more likely directly related to the availability of the androgenic ligand to its receptor, depending on the circulating levels of the ligand and potentially to the various modifiers [9]. Accordingly, heterogeneity secondary to polymorphisms in the androgen-receptor gene may contribute to the male-female disparities in severe COVID-19 cases. Apparently, ACE2 and TMPRSS2 are involved in the initial phase of the infection. The caveat of the TMPRSS2 hypothesis is that its expression, at least at the messenger RNA level, seems to be similar in male and female lungs [10]. Data regarding ACE2 expression levels in males vs females are contradictory; different studies have reported higher, similar, or lower levels of expression in males compared to females. If androgens and androgen receptors influence sex differences in mortality, it is reasonable to assume that they must affect the severity of the downstream response to the infection, including the immune response and the cytokine storm. As noted previously, females are functional mosaics of the genes on the X chromosome, which confers a stronger adaptive and immune response to viral infection compared to males.

The etiology of worse clinical outcomes in males compared to females is likely multifactorial. Understanding the differences observed in clinical outcomes of COVID-19 between men and women could prompt the consideration of a new perspective, which is linked to genetics, hormones, and sex vulnerabilities.

## Abbreviations

ACE2: angiotensin-converting enzyme II
COVID-19: Coronavirus disease 2019
PCOS: polycystic ovarian syndrome
SARS-CoV-2: severe acute respiratory syndrome coronavirus 2
*TMPRSS2*: transmembrane protease serine 2 co-receptor gene

## Additional Information

***Disclosure Summary:*** Dr Mitchell E. Geffner receives grant/research/clinical trial support from Novo Nordisk; serves as a consultant/advisory board member/advisor for Adrenas, Daiichi-Sankyo, Eton Pharmaceuticals, Ferring, Gilead, Neurocrine Biosciences, Novo Nordisk, Nutritional Growth Solutions, Pfizer, and QED; acts as a data safety monitoring board member for Ascendis, Millendo, and Tolmar; and receives royalties from UpToDate and McGraw-Hill. The other authors have nothing to disclose.
